# Editorial: Immunotherapy for Tumor in the Brain: Insights From—and For—Other Tumor Sites

**DOI:** 10.3389/fonc.2018.00128

**Published:** 2018-04-27

**Authors:** Lois A. Lampson

**Affiliations:** Brain Immunology Laboratory, Department of Neurosurgery, Brigham and Women’s Hospital, Harvard Medical School, Boston, MA, United States

**Keywords:** brain tumor, brain immunology, brain tumor immunotherapy, glioblastoma, brain metastases, immune privilege, blood–brain barrier, tumor antigen

**Editorial on the Research Topic**

**Immunotherapy for Tumor in the Brain: Insights From—and For—Other Tumor Sites**

## Tumor Immunotherapy: Common Ground

Accelerating progress in tumor immunotherapy reflects a balance between what is particular to a given tumor and what cuts across tumor types and sites, including primary and metastatic brain tumors. Although there are tumor-specific differences, the epidermal growth factor receptor (EGFR) is an important target for both glioblastoma and non-small cell lung cancer, among others, making insight into basic EGFR biology broadly relevant (1). Indirect manipulation of the immune response acts even more broadly, with checkpoint inhibitors giving durable responses against the individual antigens expressed by multiple tumor types (2). Understanding of the nature of tumor antigen is still evolving. General insights into the practical requirements for specificity (3) and the importance of neo-antigens (2) complement identification of tumor-specific targets (4).

Insights into tumor biology show a similar mix of the general and the specific. For any therapy, the eventual outgrowth of therapy-resistant tumor is common. Although resistance mechanisms vary, a general insight applies: it is now appreciated how often the potential for resistance, whether as clones with pre-existing mutations or as alternative regulatory states, is already present within the original tumor (5–8). Also appreciated is the importance of interactions between individual tumors and their local micro-environment (9–11), including those that favor immunosuppression. In this case, many details are also common, with many of the same components, such as regulatory T cells (Tregs) or cytokines (IL-10, etc.), implicated in the brain as other sites (Dutoit et al.; Perng and Lim).

## Brain Tumor: Limits and Concerns

Given these shared properties, it might be expected that tumor in the brain would show the same benefit from immunotherapy as tumor at other sites. In practice, it has been difficult to show definite benefit for brain tumor patients. This does not necessarily mean that brain tumors are more intractable. For primary brain tumors, there are practical limitations on clinical trials. The most common primary brain tumor in adults is glioblastoma (also referred to as glioblastoma multiforme or high-grade glioma). Although it’s grim prognosis gives glioblastoma prominence in public awareness and as a research focus, among all tumors it is rare (12).

Brain metastases are far more common. They are characteristic of some of the most common primary tumors, including those of the lung and breast, and of the best-studied example of successful immunotherapy, metastatic melanoma. As these and other tumors come under better control at other sites, brain metastases are increasingly important as a site of recurrence. Survival after conventional therapies for brain metastases can be just a few months, and toxicity, especially after radiation therapy, is of great concern. Despite this background, patients with brain metastases have often been excluded from clinical trials (Cohen and Kluger). Reasons have ranged from pessimism, given the poor prognosis, to specific concerns about immunotherapy.

## Rethinking “Privilege” and the Blood–Brain Barrier

A concern that has been relevant for all brain tumors, whether primary or metastatic, has been whether safe immunotherapy was even possible. A widespread, deeply entrenched assumption that the brain is, or should be, “immunologically privileged” was supported by awareness of detrimental responses, including autoimmune disease, such as multiple sclerosis, or a pathological response to neural virus. Fortunately, this concern is increasingly understood to be outdated (Huber and Irani).

From many contexts, especially work with neural viruses, it is clear that the immune response in the brain can be beneficial, is necessary, and, just as in other organs (3), is under regulatory control. Although, just as in other organs, a mis-regulated response can cause its own pathology, the immune response can safely control virus in the brain (13) (Huber and Irani; Huber et al.).

A related concern has been whether the blood–brain barrier (BBB) would prevent immune effectors from reaching brain tumor sites (14). In the normal brain parenchyma, passive entry of antibody protein is indeed blocked by the BBB. Nonetheless, antibody can affect the brain. The BBB is plastic; it changes as tumor grows. Indeed, leakage of immunoglobulin into the brain is a classic sign of pathology. The extent to which antibody can enter the brain, or accumulate, at sites of pathology, and the mechanisms by which even small amounts may be beneficial are of current interest for tumor, especially microscopic tumor (3, 14), and more broadly (15).

Although the same term is used, BBB has a different meaning for effector cells (cytotoxic T cells, etc.) than for antibodies. Metabolically active, migratory cells are well able to enter the tissues, including the brain, if appropriate signals are present (Huber et al.) (13, 16). Indeed, precursors to antibody forming *cells* can enter the brain, and make antibody from within it. Precedent is seen in the life-long production, within the brain, of antibody to neural viruses (Huber et al.) (13); this potential has not yet been intentionally exploited against brain tumor (14).

## Where We Are

A more optimistic view of immunotherapy for the brain is consistent with accumulating experience with brain tumors, as described by the papers herein. The clearest benefit has been seen for brain metastases (Cohen and Kluger). Many approaches are also being taken for glioblastoma (Ampie et al.; Dutoit et al.; Van Gool; Yamanaka and Hayano), although the work is at an earlier stage. The immune response encompasses a multitude of effector cells, molecules, and mechanisms; although broadly shared, their balance and regulation vary (3) (Huber et al.; Perng and Lim; Dutoit et al.). As targets, the biology of glioblastoma is very different from that of most metastases (3, 14); the optimal immunotherapy strategy need not be the same.

Today, we have seen that the immune response is able to control human tumor, and that the response can be intentionally enhanced (2). The field is young. Not every patient benefits, many tumors recur, and often responses are not well-controlled. The papers herein illustrate growing appreciation that immunotherapy, its potential and its challenges, are just as relevant for the brain as for other sites. Achieving a balance between immunotherapy and autoimmunity is a general challenge, not only for tumors, and has been a special concern for the brain. As the brain’s relevance becomes accepted, insights gained from the brain and other sites should reinforce each other.

## Author Contributions

The author confirms being the sole contributor of this work and approved it for publication.

## Conflict of Interest Statement

The author declares that the editorial was written in the absence of any commercial or financial relationships that could be construed as a potential conflict of interest.
